# Salivary Biomarkers in Periodontitis Patients: A Pilot Study

**DOI:** 10.1155/2022/3664516

**Published:** 2022-03-24

**Authors:** Sarah Reddahi, Amal Bouziane, Sana Rida, Houssain Tligui, Oumkeltoum Ennibi

**Affiliations:** ^1^Research Laboratory in Oral Biology and Biotechnology, Faculty of Dental Medicine, Mohammed V University in Rabat, Rabat, Morocco; ^2^Department of Periodontology, Faculty of Dental Medicine, Laboratory of Biostatistics, Clinical Research and Epidemiology, Mohammed V University in Rabat, Rabat, Morocco; ^**3**^ Department of Restorative Dentistry, Research Laboratory in Oral Biology and Biotechnology, Faculty of Dental Medicine, Mohammed V University in Rabat, Rabat, Morocco; ^**4**^ Medical Research Laboratory, Children's Hospital, University Centre Ibn Sina, Faculty of Medicine and Pharmacy, Mohammed V University in Rabat, Rabat, Morocco; ^**5**^ Department of Periodontology, Research Laboratory in Oral Biology and Biotechnology, Faculty of Dental Medicine, Mohammed V University in Rabat, Rabat, Morocco

## Abstract

**Materials and Methods:**

Forty subjects were included: 10 periodontally healthy subjects and 30 periodontitis patients. Periodontal examination and saliva sampling were performed in all patients. Levels of salivary cytokines including IL-1*β*, IL-6, MMP-8, and IL-10 were evaluated by a sandwich ELISA test kit. Data were analyzed by SPSS for Windows.

**Results:**

Regarding individual biomarkers, IL-1*β*, IL-6, and MMP-8 levels were significantly higher in periodontitis patients (*p* ≤ 0.001, *p* < 0.05, respectively). The concentration of these proteins in saliva showed a significant association with gingival index and pocket depth measurements and may reflect the clinical status of healthy and diseased periodontium. However, no significant differences were observed for the IL-10 component.

**Conclusion:**

IL-1*β* and IL-6 concentrations were statistically higher in periodontitis patients and may be used as potential tools in periodontitis diagnosis.

## 1. Introduction

Periodontitis is a multifactorial inflammatory chronic disease initiated by a dysbiosis of the commensal oral microbiota. Its pathogenesis is related to a complex interaction between periodontal bacteria (such as *Porphyromonas gingivalis*, *Tannerella forsythia, Fusobacterium nucleatum*, and *Aggregatibacter actinomycetemcomitans*) and the host inflammatory cell-mediated immune response, and if exaggerated, this phenomenon may lead to the deeper periodontal tissue destruction [1–3]. Many other factors, including increasing age, local factors, environmental factors, behavioral habits (smoking, stress, and diet), chronic systemic diseases such as diabetes mellitus, and genetics are involved in the etiology of the disease. [4–6]. To remain healthy, the periodontium and the host should be able to adapt to changes that may occur in the presence of the above factors. Periodontal homeostasis and changes are also regulated by genetics. Indeed, it is unanimously accepted that genes encode both immune receptors and molecules that influence the specificity and sensitivity of the host to bacterial species. This phenomenon regulates the intensity of the inflammatory response by encoding and adapting the signal transduction pathways upstream and downstream of the inflammatory signals, allowing flexible response of the periodontium to external and internal stimuli [7–9].

The current diagnosis method of periodontal diseases is based mainly on radiographic and clinical examinations of periodontal tissues. However, tools for early detection, evaluation of severity, and prognosis of periodontal disease are presently insufficient. Based on this, disease-specific biomarkers in oral fluids as a complement to regular clinical parameters are of interest. It is important to mention that several biomarkers can be detected in the gingival crevicular fluid (GCF) and saliva. Such biomarkers can be fundamental for the determination of the levels of inflammatory mediators released during periodontal disease progression [10, 11].

Activated proinflammatory cytokines in the gingival tissues, as part of the innate response of resident cells (e.g., epithelial cells and/or fibroblasts) and neutrophils, are responsible for the generation of disproportionate amounts of inflammatory mediators, including cytokines-chemokines and matrix-metalloproteinases, that stimulate soft and hard tissue destruction [12].

Many studies have raised an important association between cytokines, originated from neutrophils and other defense cells, and different stages of periodontal disease, especially interleukin 1-*β*, IL-6, tumor necrosis factor-*α* (TNF-*α*), and receptor activator of nuclear factor kappa-B ligand (RANKL), that seem to have the most substantial role as central key elements in the cytokine networks of the development of periodontal diseases [13, 14]. Also, C-reactive proteins (CRP), IL-12, MMP-8, MMP-9, and MMP-13 have been described as biomarkers for the periodontal disease [15–17].

Saliva, an oral fluid rich in proteins and genetic molecules, has been used in the diagnosis of a number of systemic diseases and immunological and nutritional evaluations, and it is also commercially available through many tests [18, 19]. Whole saliva is a mixture of liquid products from major and minor salivary glands, and it also contains components of gingival crevicular exudates, oral bacteria, and their products, proteins, glycoproteins, electrolytes, and small organic molecules, such as compounds originating from blood. In recent years, saliva is often used for diagnostic purposes [20–22]. These properties of saliva open the door to a perfect method of health exploration and disease surveillance in clinical settings with a minimal amount of oral fluid, since it is considered to be an easily available, noninvasive diagnostic tool. Although the molecules in saliva may not provide special information relating to specific site conditions, they have been utilized to follow changes concerning the whole mouth [23]. A number of promising biomarkers have been identified as being potential salivary biomarkers for periodontal disease [24].

Periodontitis is known as the sixth most common chronic inflammatory disease in the world [25] that occur in more than 50% of adults worldwide [26]. The rate of the disease differs with ethnicity and genetic polymorphism [27]. Moreover, according to recent systematic reviews and meta-analyses, salivary IL-1*β*, IL-6, and MMP-8 are among the five promising biomarkers that have been identified as eligible candidates for the diagnosis of periodontal diseases [28, 29]; while IL-10 may have a strong regulatory effect on immune responses [30]. However, disparate findings were reported in different studies that were held mainly in the Caucasian and Asian populations.

Our hypothesis was that salivary levels of biomarkers in subjects with periodontitis might differ according to ethnicity and genetics. Thus, the aim of the present study was to evaluate salivary concentrations of IL-1*β*, IL-6, MMP-8, and IL-10 in healthy and periodontitis patients and to assess the association between these biomarkers levels and clinical parameters in a Moroccan population.

## 2. Materials and Methods

### 2.1. Study Population

**Figure 1:**
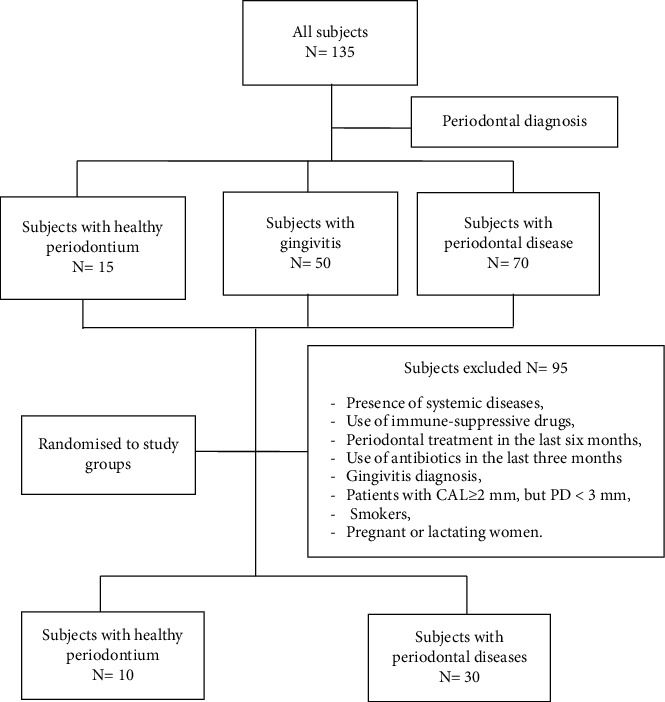
Flowchart of the progress of subjects through the phases of the study.

The study was designed as a descriptive and analytic cross-sectional study. Forty subjects who participated in this trial were randomly selected from patients seeking periodontal treatment at the Department of Periodontology in the Center of Dental Consultation and Treatment (CCTD) Ibn Sina' University Hospital Center (CHUIS), from March 2019 to February 2020. According to their periodontal status and general health condition, eligible patients were invited to take part in the study (Figure 1).

The inclusion criteria for healthy group were systemically healthy subjects aging 18 years old and above, nonsmokers, and individuals having no less than 20 teeth. Exclusion criteria included the presence of any systemic chronic disease (including diabetes, obesity, hypertension, heart disease, liver disease, malignancy, and autoimmune diseases), oral mucosal diseases, use of antibiotic treatment during the 6 months preceding the clinical examination, periodontal treatment received during the past six months, and pregnant or lactating woman.

The Ethical Committee for Biomedical Research at Mohammed V University Faculty of Medicine and Pharmacy of Rabat approved the study protocol (No. 62/18). Each participant was given verbal and written information that described the nature of the study. Furthermore, the participants signed an informed consent form written in the native language in accordance with Helsinki Declaration.

### 2.2. Saliva Sampling

Salivary sampling was performed from 08 : 30 am to 11 : 30 am. Participants were asked to avoid eating, drinking, and brushing their teeth in the morning prior to examination. First, each participant took a seat and gargled with tap water. Then, they had to expectorate entire saliva in a small sterile cup for 5 minutes using the drooling technique. Following that, 2 to 5 ml of unstimulated saliva sample was transferred to a polypropylene tube. Immediately, collected saliva samples were centrifuged for 15 minutes (6000 rpm, 4°C), then distributed in Eppendorf tubes and frozen at −80°C until analysis.

### 2.3. Periodontal Examination

All clinical measurements were performed by two calibrated examiners (E.OK and B.A). An overall interexaminer reproducibility, as evaluated by intraclass correlation coefficient, was 0.85 to 1 for clinical attachment loss and periodontal pocket depth measurements.

All clinical findings were recorded on data collection worksheets, including plaque index (PI) using dichotomous scoring (O'Leary Index), bleeding on probing (BOP), sulcus/probing pocket depth (PPD) measurements, and clinical attachment loss (CAL). Probing depths and attachment loss were measured at six sites (distobuccal, buccal, mesiobuccal, distolingual, lingual, and mesiolingual) for each tooth, excluding third molars, using a standardized North Carolina periodontal probe (Hu-Friedy, Chicago, IL, USA). Probing pocket depths were assessed from the edge of marginal gingiva to the bottom of the pocket, and attachment loss is assessed from the cementoanemal junction to the bottom of the pocket. Supported by a radiographic examination, an initial diagnosis was made after recording the measurements mentioned above. A healthy periodontium was defined as the absence of either gingivitis or periodontitis history (BOP < 10%, PPD ≤  3 mm, no proximal clinical attachment loss (CAL), and no recession).However, a periodontitis patient was defined as a patient who had at least two nonadjacent teeth with interdental clinical attachment loss (CAL) ≥ 2 mm, probing pocket depth (PPD) > 3 mm, and radiographic evidence of bone loss [31, 32].

Periodontitis patients were classified by stage and grade according to the new classification proposed by the 2017 International World Workshop. Definitions of stages were based on severity (primarily periodontal breakdown and periodontitis-associated tooth loss), complexity of management (pocket depth, infrabony defects, tooth mobility, furcation defects, masticatory deficiency), and grade definitions of periodontitis were based on direct or indirect evidence of progression rate of the disease. Three categories can be distinguished as follows: slow (Grade A), moderate (Grade B), and rapid rate of progression (Grade C) [32].

### 2.4. Salivary Molecular Analysis

The INVITROGEN Sandwich ELISA kit with a precoated plate was used for the detection of IL-1*β*, IL-6, MMP-8, and IL-10. Each kit was used to analyze individual half diluted saliva samples (by mixing 50 *μ*l of saliva with 50 *μ*l of sample diluents buffer) for the four potential biomarkers according to the manufacturer's instructions. The wash steps were performed automatically by using an ELISA plate washer. All analyses were performed in duplicate. Briefly, standards and half diluted saliva samples were added in respective mono anti-cytokine antibody precoated wells and incubated for 2 hours. After the plates were washed, the biotin-conjugated detection antibody was added. After another 60 min of incubation and subsequent washing, streptavidin-conjugated PE was added for 30 min. After an additional wash, the complex was solubilized by adding the Bio-Plex assay buffer to each well and kept in the dark for another 30 min. Then, plates were analyzed using an ELx800 Absorbance Microplate Reader by BioTek Instruments, Inc., Vermont, the USA, at a wavelength of 450 nm. For all assays, results were measured by the intensity of the signal produced by the enzyme-antibody-target complex that is directly proportional to the concentration of target present in the original sample. Total amounts (pg/ml) of each cytokine were determined using the standard curves created in each assay according to the kit protocol. The detection limits for analyzed cytokines and MMP-8 enzyme were as follows: IL-1*β* 0.9 pg/ml and a coefficient of variation of 4.7%, IL-6 0.9 pg/ml and a coefficient of variation of 3.4%, IL-10 2.7 pg/ml and a coefficient of variation <10%, and MMP-8 had a detection limit of 6 pg/ml and a coefficient of variation <10%.

### 2.5. Statistical Analysis

Data were analyzed using SPSS for Windows 20.0. Descriptive statistics were expressed as mean standard deviation or median and interquartile range (IQR) for the quantitative variables and as frequencies and percentages for the qualitative variables. To evaluate the relationship between different parameters among the studied groups, Pearson's chi-square test was used for the qualitative variables and the Mann–Whitney test was used for the quantitative results. The Kruskal–Wallis test was performed to compare biomarkers concentration between the different groups. The statistical significance level used was *p* ≤ 0.05.

## 3. Results

Forty randomly selected subjects were recruited for the study according to inclusion and exclusion criteria (Figure 1). Thirty patients having a periodontal breakdown were assigned to periodontitis group and ten periodontally healthy subjects were assigned to the nonperiodontitis group.

The demographic characteristics of the studied population are shown in Table 1. There were no significant differences in age and gender between periodontitis and healthy groups (*p*=0.131 and *p*=0.206, respectively). However, there were statistically significant differences regarding PI, BOP, and pocket depths in diseased group compared to healthy subjects (*p* <  0.001).

Significant higher salivary levels of IL-6 and IL-1*β* were found in the periodontitis group when compared to the nonperiodontitis group (*p*=0.005; *p* <  0.001, respectively). Regarding MMP-8, despite being higher in concentration in periodontitis subjects compared to the healthy subjects, the difference between the groups was not statistically significant (*p*=0.059). There was no statistically significant difference in IL-10 levels (*p* > 0.05) (Table 2).

The periodontitis group was analyzed regarding the severity and the possible rate of progression.  Table 3 summarizes the clinical characteristic in periodontitis group grades B and C. The median (IQR) scores of PI, BOP, PD, and CAL parameters in the diseased group with B grade and C grade showed no statistically significant differences. Grade C periodontitis patients had significantly higher periodontal probing >3 mm score and higher number of sites with PD ≥ 7 mm and CAL ≥ 5 mm than B grade periodontitis group, respectively, (*p*=0.029, *p*=0.050, and *p*=0.018).

Inflammatory markers measurement in saliva was considered for the entire study population. The comparison between the 3 different groups presented in Table 4 showed a significantly higher level of IL-1*β* in the grade B and the grade C periodontitis groups than those in the healthy control (HC) group (*p* < 0.001). The IL-6 level was significantly higher in the grade C group than in the HC group (*p* < 0.05). MMP-8 saliva concentrations in diseased groups were higher than in the healthy group; however, there were no statistically significant differences between them (*p*=0.057). No significant differences were observed for the IL-10 component, where it was globally absent among all our population categories (Table 4).

## 4. Discussion

The present study analyzed salivary levels of IL-1*β*, IL-6, MMP-8, and IL-10 in periodontally healthy subjects and stage III periodontitis individuals. Age distribution was not significantly different between the groups (*p* > 0.05). This could be explained by the age range of the participants, which was quite small (Table 1). However, in diseased subgroups, patients with periodontitis stage III grade B were older compared to patients with periodontitis stage III grade C, and the difference was statistically significant. Indeed, periodontitis grade C, formally known as aggressive periodontitis, occurs most likely among young adults, which was the case in this study [32, 33]. Regarding gender, no statistical differences were found, as previously reported by many studies [34, 35].

Patients with periodontitis stage III grade B and grade C had statistically higher levels of IL-1 and IL-6 than healthy participants (*p* < 0.05) (Table 3 and 4).

IL-1*β* is a proinflammatory cytokine recognized as an important mediator in the pathophysiology of periodontitis. Gursoy et al. suggested that IL-1*β* is a well-potent inflammatory stimulator that can help discriminating between inactive and active periodontal lesions [36]. Its properties include promoting bone resorption and inducing the production of tissue-degrading proteinases [37]. Our findings of higher IL-1*β* concentrations in saliva from patients with grade B and C periodontitis are in agreement with several earlier studies that showed higher salivary levels of this analyte in patients suffering from periodontitis in comparison with healthy subjects [38–42]. It has been reported that patients with deeper pocket depths and more severe bleeding on probing (BOP) had increased value levels of salivary IL-1*β*. Clinical evidence has shown that periodontal disease lead to excessive release of IL-1 *β*, with a sensitivity ranging from 54% to 88% and a specificity ranging from 52% to 100% across five studies [27, 28, 39, 43, 44]. The authors of [35] evaluated salivary interleukin IL-1*β*, matrix metalloproteinase MMP-8, and pyridinoline cross-linked carboxyterminal telopeptide of type I collagen (ICTP) as potential diagnostic tools for periodontitis diagnosis. They concluded that as a single marker IL-1*β* showed the best diagnostic value with 90% sensitivity and 76% specificity for discriminating periodontitis subjects from healthy subjects. Thus, IL-1*β* is considered to be a strong promising biomarker for the early diagnosis of periodontal disease.

Il-6 was also assessed in many studies as a proinflammatory cytokine acting on bone destruction in presence of infection [41]. In our study, the IL-6 level was significantly higher in grade C periodontitis group than the healthy group (*p* < 0.005) (Table 4). These results are consistent with previous findings. Indeed, it has been suggested that salivary IL-6 concentration increased significantly in periodontitis patients as compared to healthy controls and these levels increased with the progression of the disease [45]. The authors of [46] reported a significant proportional increase of salivary IL-6 levels with a proportional increase in clinical attachment loss, periodontal probing depth, and bleeding on probing. However, the authors of [27] did not find any statistical difference in the IL-6 level value is saliva samples from Taiwanese population.

Many studies have examined one or more inflammatory salivary markers to be used as target molecules for periodontal disease diagnosis and showed significantly different concentrations of the studied cytokines between diseased and healthy subjects. However, up to now there has been no clear and convincing biomarker that can be used for diagnosing periodontitis. The present study aimed to evaluate 3 biomarkers (i.e., IL-1*β*, IL-6, and MMP-8) out of the 5 most promising salivary host-derived biomarkers identified by recent systematic reviews and meta-analyses as good candidates to be chosen for the early diagnosis of periodontitis [29, 47].

Although there was no significant statistical difference in MMP-8 levels between the diseased and healthy groups (*p*=0.057), the concentration was higher in the diseased group. The lack of significant association may be related to the small size of the population mainly in the healthy group. Early studies showed that MMP-8 levels were statistically higher in periodontitis patients compared to nondiseased ones [34, 42, 48].

Metalloproteinase-8 (MMP-8), released by neutrophils, is a molecule having the unique ability to break down type I and III collagens, which are the major collagen species within the periodontium [41]. Many authors have reported MMP-8 as one of the strongest markers for tissue destruction and as a promising biomarker for periodontal early diagnosis with sensitivity ranging from 65% to 87%, and specificity ranging from 48% to 87% [28, 39, 47, 49]. High levels of MMP-8 in saliva were associated with all clinical periodontal parameters, namely, probing depth, clinical attachment loss, BOP, and PI. The authors of [50] reported an increased concentration of salivary levels of MMP-8 in patients with generalized moderate to severe periodontitis. Some studies reported that no statistically difference could be found between gingivitis and healthy periodontium regarding saliva levels of MMP-8 [51]. The authors of [52] showed that total MMP-8 may not be able to reflect effectively periodontal breakdown or progression of periodontitis and they stated that instead of total MMP-8, the assessment of active MMP-8 (aMMP-8) levels may reflect a proinflammatory state of periodontal disease and may help in staging and grading periodontitis.

The authors of [53] reported that if proinflammatory cytokines, including IL-1*α*, IL-1*β*, TNF-*α*, IL-6, and IL-17 contribute to severe chronic inflammation and tissue breakdown, other cytokines such as IL-10 have antagonist effects and may help on regulating immune response. In this study, we included also IL-10 as an anti-inflammatory or immunosuppressive mediator that may exert potent regulatory effects on immune response in periodontal disease.

IL-10 released by T helper 2 (Th2) is an anti-inflammatory and immunosuppressive cytokine. It has the capacity to inhibit cytokine synthesis by T cells which may deploy a strong regulatory effect on immune responses in periodontal disease. It is less seen or absent in severe periodontitis [54, 55], which corroborate with our results. Many studies reported the positive role of IL-10 in preventing periodontitis progression and promoting its stability. Previous in vitro studies reported that IL-10 seems to inhibit osteoclast cells [56] and can downregulate IL-1*β*, TNF-*α*, and IL-6 secretion [57, 58]. IL-10 intervenes in tissue homeostasis as well [59]. Thus, high saliva levels of IL10 can be more accurate after periodontal treatment.

Salivary analytes are biological reflections of inflammatory and tissue destructive processes during periodontitis, and some of them can be used as tool to “intercept” early stages of periodontal diseases. Many studies reported that rather than considering a unique salivary biomarker for periodontitis diagnosis, assessing a panel of biomarkers may show more effective interest in periodontitis diagnosis [60]. The authors of [39] reported that a panel consisting of IL-1*β*, MMP-8, and IL-6 shows particular diagnostic interest for periodontitis. Among 10 studied salivary biomarkers, the authors of [27] found that IL-1*β*, MMP-8, and MMP-9 showed a good potential for identifying patients with periodontitis. Moreover, the combination of IL-1*β* and MMP-8 can be used to discriminate gingivitis subjects from healthy subjects [35].

According to some systematic reviews, our studied salivary molecules, IL-1*β*, IL-6, and MMP-8 are recognized as key salivary biomarkers with acceptable diagnostic reliability for periodontal disease [29, 49]. When developing the new framework for staging and grading in the case definition system of periodontitis, the teamwork suggested that specific biomarkers and their thresholds may be incorporated in diagnostic criteria. Indeed, validated biomarkers may help improving a periodontitis diagnosis notably in the early stages, and probably also to assess disease development and clinical response to treatment in an otherwise healthy individual with high risk to develop periodontitis [61].

To our knowledge, this study is the first one looking for salivary biomarkers in patients with periodontitis in an African country. Results about presence of cytokines in periodontitis may show some discordance between studies. This could be due to studies protocols, sample size of the studied groups, and also because periodontitis are chronic diseases with dynamic states of activity and remission. Moreover, geographic and ethnic disparity may have a potential role in differences between data. Indeed, beside dysbiosis and host response, multiples factors may be involved in development and worsening of periodontal diseases including aging factor, low economic status, smoking, genetics, and geographical factors [62, 63]. Thus, more investigations are necessary to clarify to reel potential of a single biomarker or a panel of biomarkers to be associated with periodontitis diagnosis, to be associated with periodontitis development, or even to be used as a tool to assess periodontal response to treatments.

There were several limitations to this study. First, even if we were able to detect statistically significant differences in IL-1 and Il-6 levels between the diseased and healthy groups, the sample size was likely insufficient to detect the true differences in MMP-8 and IL-10 levels among the studied population. In these short time data, patients with gingivitis or mild periodontitis were not included. The association of biomarker levels and disease severity was therefore difficult to assess. These data add to previous findings that might be used together with future studies targeting specific biomarkers and their thresholds, which could be incorporated into the clinical diagnosis of periodontitis.

## 5. Conclusion

Patients with periodontal disease had significantly higher levels of IL-1 and IL-6 in their saliva than those with healthy periodontium. Despite the limitations of our study, we believe that these biomarkers could be useful diagnostic tools for periodontitis. However, screening a broader sample in a longitudinal study could help determine how accurate these biomarkers are at predicting periodontitis progression in the Moroccan patients with periodontitis.

## Figures and Tables

**Table 1:** Demographics and clinical characteristics of the population.

	Healthy periodontium subjects (n = 10)	Patients with periodontal disease (n = 30)	*p* value
Age^a^	24.80 ± 2.04	29.83 ± 10.16	0.131
Gender^b^			0.206
Male	1 (10)	9 (30)	
Female	9 (90)	21 (70)	
Periodontal indices			
PI (%)	5.26 ± 1.97	95.4 ± 8.01	<0.001
BOP (%)	3.75 ± 2.45	93.75 ± 9.0	<0.001
PD^a^	2.44 ± 0.24	7.01 ± 1.44	<0.001
CAL^a^	0	3.85 ± 1.41	NA
Number of sites with PD ˃3mm^a^	0	5.53 ± 0.86	NA
Number of teeth^a^	26.43 ± 2.97	27.7 ± 0.67	0.595

^a^Mean ± SD; ^b^n (%). PD: probing depth; CAL: clinical attachment level; PI: plaque index score; BOP: bleeding on probing scores; NA: not applicable.

**Table 2:** Salivary levels of IL-6, IL-1*β*, IL-10, and MMP8 in nonperiodontitis and periodontitis patients.

Average concentration of studied cytokines	Nonperiodontitis group N = 10	Periodontitis group N = 30	*p*
IL-6 (pg/ml)	0.00[0.00–0.00]^*∗*^	0.38[0.00–1.00]	0.005
IL-1*β* (pg/ml)	0.00[0.00–0.00]^*∗*^	11.25[8.00–15.00]	<0.001
MMP-8 (pg/ml)	431.50[187.00–1355.00	1150.00 [823.75–1603.75	0.059
IL-10 (pg/ml)	0.00[0.00–0.00]^*∗*^	0.00[0.00–2.63]	0.092

Data are presented as median (interquartile range). Independent *t*-test was conducted to compare the difference of biomarkers levels between two groups. ^*∗*^Levels under the threshold limit.

**Table 3:** Periodontal clinical parameters in the periodontitis group.

	Periodontitis patients Grade B n = 5	Periodontitis patients Grade C n = 25	*p*
Age^a^ (mean+/- SD)	42.63 ± 3.81	25.00 ± 6.57	<0.001
Plaque index^b^	100 [100–100]	100 [100–100]	0.75
Bleeding on probing^b^	100 [100–100]	100 [100–100]	0.85
Mean periodontal pocket depth^b^	4 .66 [4.02–5.02]	4.04 [3.39–5.03]	0.25
Periodontal probing >3 mm	4.96 [4.46–5.22]	5.56 [5.17–6.47]	0.029
Clinical attachment loss^b^ (mm)	3.02 [2.65–3.44]	4.14 [3.02–4.81]	0.056
Number of sites with PD ≥ 7 mm^b^	1.00 [0.00–3.25]	5.00 [2.00–12.50]	0.047
Number of sites with CAL^b^ ≥ 5 mm	7.00 [1.75–12.75]	17.00 [5.75–32.75]	0.018

^a^Mean ± SD; ^b^median and interquartiles. The Mann–Whitney test was used to evaluate the difference between the two groups.

**Table 4:** Salivary mediator levels

	HC (n = 10)	Grade B periodontitis (n = 5)	Grade C periodontitis (n = 25)	*p*
IL-1*β* (pg/mL)	0.00 [0.00–1.38]	8.50 [6.00–12.75]^*∗*^	12.25 [8.00–21.63]^*∗*^	<0.001
IL-6 (pg/mL)	0.00 [0.00–0.00]	0.00 [0.00–1.00]	0.50 [0.00–1.50] †	0.013
MMP-8 (pg/mL)	431.50 [182.00–1385.00]	1257.50 [805.00–1617.50]	1125.00 [803.75–1630.00]	0.057
IL-10 (pg/mL)	0.00 [0.00–0.38]	1.50 [0.00–3.00]	0.00 [0.00–1.88]	0.155

Data summarized as median and interquartile range (IQR); *p* value derived from the Kruskal–Wallis test and Mann–Whitney *U* test. ^*∗*^Significant difference from the HC group (*p* ≤ 0.01). ^†^Significant difference from the HC group (*p* < 0.05). HC: healthy control.

## Data Availability

All data used to support the findings of this study are included in the manuscript.
